# Projected and historical amplification of moisture fluxes towards Antarctica by synoptic eddies

**DOI:** 10.1038/s41612-026-01376-x

**Published:** 2026-04-02

**Authors:** Patrick Martineau, Hua Lu, Thomas J. Bracegirdle

**Affiliations:** 1 Japan Agency for Marine-Earth Science and Technology, Yokohama, Japan; 2 British Antarctic Survey, Cambridge, UK

**Keywords:** Climate sciences, Environmental sciences

## Abstract

Synoptic weather systems play a crucial role in transporting moisture to Antarctica. Climate models project significant changes in these systems, including a wintertime intensification and a summertime poleward shift, with implications for Antarctic ice mass balance. Our analysis of CMIP6 model output shows synoptic moisture fluxes across the Antarctic Circle increasing by 2–6% per decade under high-emission scenarios, accounting for 24% of winter and 93% of summer total moisture transport trends. This increase is mainly associated with enhanced eddy moisture anomalies rather than stronger eddy wind anomalies that are often used to gauge storm track activity. Eddy-driven moisture variability also accounts for a substantial fraction of inter-model uncertainty in future projections. Furthermore, using a large-ensemble approach, we show that differences between reanalysis and multi-model mean externally forced trends could possibly be due to natural climatic variability, while potential model biases cannot be excluded.

## Introduction

Atmospheric water vapour transport plays a critical role in regulating the mass balance of the Antarctic ice sheet by producing precipitation^1–4^. Given the limited moisture sources over Antarctica, most of the precipitation is attributed to moisture transported from lower latitudes by the atmosphere. This moisture originates mainly from the equatorial flank of the Southern Ocean, where oceanic evaporation acts as an important moisture source for the overlying atmosphere^1,5^. The moisture is then primarily transported towards Antarctica in the lower troposphere^6^^,^^7^^,^ through transient weather systems^8–11^.

Transport across the Antarctic circle (~66.5°S) predominantly occurs through low-pressure systems developing in the circumpolar trough^12^. These systems are characterized by synoptic-scale cyclonic eddies^13,14^ and mesoscale frontal systems^14,15^. As these low-pressure systems develop in the surrounding regions and advance towards Antarctica, they transport moisture from the lower latitudes^16–18^, thereby contributing to precipitation across the continent.

Extreme events such as atmospheric rivers (ARs), long and narrow filament-shaped structures that carry abundant moisture from the mid-latitudes or even subtropics, strongly contribute to moisture fluxes towards Antarctica^19–22^. The ARs typically align with strong cyclones situated to the west of their centers, as well as with anticyclones positioned to the east^23,24^. Those ARs that make landfall in Antarctica tend to be steered by blocking high-pressure systems that contribute to enhancing their stationarity^22^.

Synoptic weather systems, encompassing the various aforementioned moisture transport mechanisms, are undergoing profound changes under global warming. A general intensification of synoptic activity has been detected year-round in reanalysis data^25–28^ as evidenced by diagnostics of eddy kinetic energy (EKE), heat fluxes, and meridional moisture fluxes. In summer, a poleward shift is observed in addition to the intensification^25^. While earlier studies did not identify significant trends in moisture transport towards Antarctica^8,29^, more recent findings suggest that this intensification of synoptic activity is accompanied by a robust enhancement of poleward moisture fluxes near the Antarctic circle^25^.

In broad agreement with recent trends, synoptic activity is projected to undergo profound changes according to climate models from the fifth and sixth phases of the Coupled Model Intercomparison Project (CMIP5 and CMIP6). Notably, both CMIP5 and CMIP6 models project a wintertime intensification and summertime southward shift of the Southern Hemisphere (SH) storm track^26^, under both high and moderate greenhouse gas emission scenarios^30,31^. These trends are commonly diagnosed using metrics such as vertically integrated EKE and meridional wind variance. While these metrics do not directly quantify moisture fluxes, their trends suggest potential changes in moisture transport to Antarctica. In winter, intensification trends have been identified using both hemispheric-mean EKE and moist static energy^26^.

Despite inter-model variability, an overall intensification of Antarctic precipitation has been projected by all CMIP3 simulations^32^. The projected increases are consistently attributed to thermodynamic processes, rather than changes in atmospheric circulation. Across CMIP3 models, enhanced precipitation arises primarily from the greater moisture-holding capacity of a warmer atmosphere, which amplifies precipitation under similar synoptic conditions. Another study of future precipitation trends based on atmospheric general circulation models forced by CMIP3-derived sea surface temperatures and ice concentrations also confirms the dominant role of thermodynamic forcing^33^. Scaling analyses further show that changes in high-frequency, vertically integrated moisture fluxes are strongly linked to thermodynamic factors^34^. More recent simulations from CESM2 (CMIP6), CMIP5 models, and the CESM large ensemble project similarly indicate that increased snow accumulation over Antarctica is largely explained by thermodynamic mechanisms, with sub-monthly weather variability playing a key role^35^. However, whether these findings hold for the latest generation of IPCC models remains uncertain.

To better understand factors influencing future climate projections of moisture transport towards Antarctica, here we delve into the repercussions of anticipated changes in synoptic eddy activity on this transport. This is achieved through an analysis of both historical and future climate change scenarios from CMIP6^36^ as well as reanalysis records. Specifically, we aim to address the following questions: How do historical trends in eddy-driven moisture transport, as modelled by CMIP6, compare with those derived from reanalysis products? Have externally forced trends emerged from natural variability in reanalysis data? How is poleward moisture transport by synoptic eddies projected to change in a warming climate? What roles do thermodynamic and dynamic factors play in driving changes in poleward moisture flux? How consistent are these projected changes across CMIP6 models?

## Results

### Hemispheric patterns of synoptic moisture transport trends

**Fig. 1:**
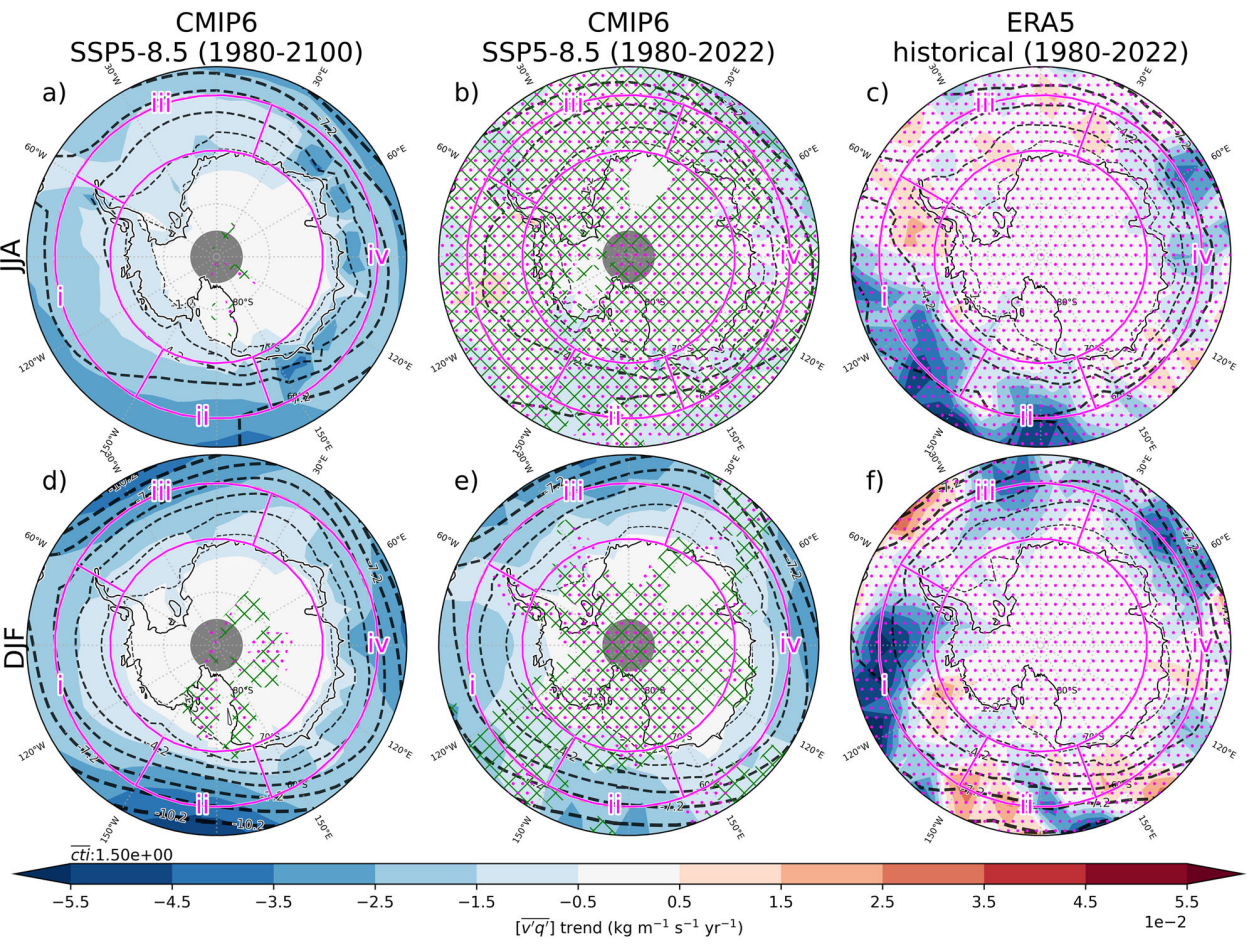
Spatial distribution of trends in meridional moisture flux. Vertically-integrated synoptic meridional moisture flux $([\overline{{v}^{\mathrm{'}}{q}^{\mathrm{'}}}])\,$ trends (1980-2022; kg m^−1^ s^−1^ yr^−1^) are contrasted between (middle column) CMIP6 models under SSP5-8.5 and ERA5 (rightmost column). The same trends are also assessed in CMIP6 models for an extended period (1980–2100; leftmost column). They are shown separately for (top; **a**), **b**), **c**)) JJA and (bottom; **d**), **e**), **f**)) DJF. Trends are illustrated with colour shadings and the climatological reference for the same period is illustrated with black contour lines at 1.5 kg m^−1^ s^−1^ intervals (dashed for negative [southward] fluxes) with thicker contours for larger magnitudes. Sectors where less than 2/3 of the models agree on the sign of the trend are stippled in green. The false discovery rate method^53^ is used to assess significance in ERA5 and CMIP6 models with a false discovery control value (αFDR) of 0.1 (see methods). Non-significant trends are indicated with magenta stippling. Sectors over which area averages are later performed are contoured in magenta.

To place Antarctic moisture flux trends in a broader hemispheric context, Fig. 1 presents the spatial distribution of vertically integrated moisture flux $([\overline{{v}^{\mathrm{'}}{q}^{\mathrm{'}}}])$ trends across the SH extratropics for both winter (June-July-August; JJA) and summer (December-January-February; DJF). Results are presented for the short term (1980–2022) and the long term (1980–2100), enabling comparison of historical trends from CMIP6 models and reanalyses, as well as evaluation of projected future changes. Here, the CMIP6 multi-model is computed by first averaging moisture fluxes seasonally and then across datasets, with trends subsequently assessed from the resulting mean time series. We note that the fluxes are climatologically negative (i.e., poleward), such that a negative trend indicates an amplification of moisture transport toward Antarctica. The trend in long-term (1980-2100) CMIP6 projections is predominantly negative and statistically significant throughout almost the entire SH high latitudes in both JJA and DJF. Trends increase in magnitude towards the equator, where synoptic eddies typically transport more moisture southward, and decrease progressively in amplitude towards the pole, where water vapour becomes increasingly scarce. Larger trends are found over East Antarctica in JJA. In DJF, a zonal wavenumber 3 trend pattern is found to amplify the climatological pattern of the same scale in the midlatitudes with maxima at around 30°W, 170°W, and 90°E, bearing resemblance with stationary waves observed in the geopotential height field^37^. While modes of wavenumber 3 variability of the 500-hPa height field do not exhibit significant trends in CMIP6 projections^38^, a clear wavenumber 3 pattern is observed in sea level pressure trends^39^ which could be related to the modulation of synoptic moisture fluxes.

When assessing short-term (1980–2022) trends, the JJA simulated trends are markedly less pronounced than their long-term counterpart and not statistically significant. In contrast, during summer, the pattern of externally-forced simulated trends closely resembles that of the longer period, albeit slightly weaker. In both seasons, the externally-forced trends in CMIP6 models are markedly different from the historical evolution seen in ERA5, where they are notably less zonally symmetric. This highlights the important role of internal variability in the observed trends, a point which will be examined in greater detail in subsequent sections.

### Longitudinal structure of moisture flux changes along the Antarctic Circle

**Fig. 2:**
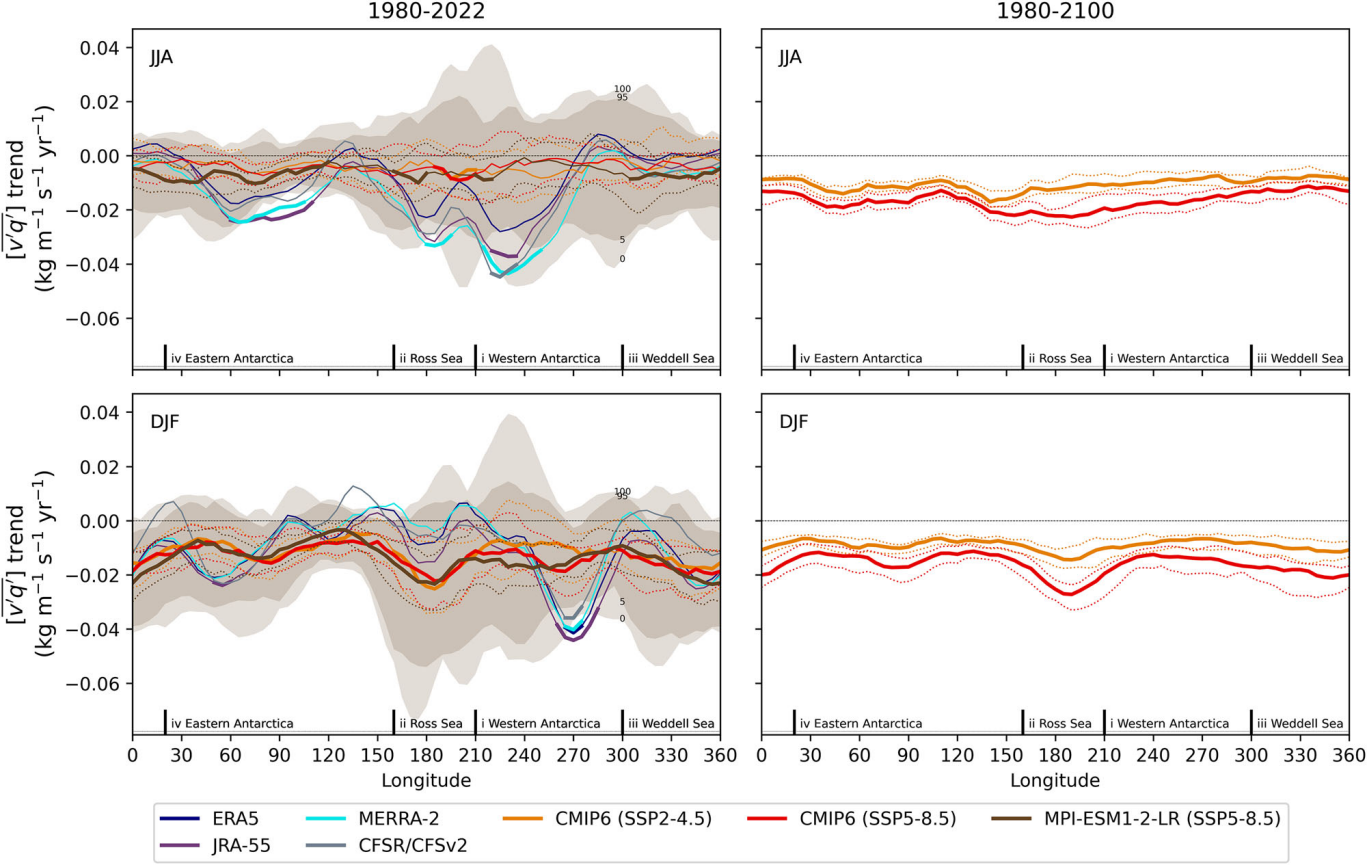
Zonal profile of moisture transport trends. Vertically-integrated synoptic moisture flux trends ($(\overline{{v}^{\mathrm{'}}{q}^{\mathrm{'}}})$; kg m^−1^ s^−1^ yr^−1^), averaged over 60–70°S, are shown for reanalysis datasets and CMIP6 models for (top) JJA and (bottom) DJF, separated into (left) short-term and (right) long-term periods. Solid lines indicate the trends from reanalyses and the median trend among models, while dotted lines represent the interquartile range (25th–75th percentiles). The 0–100% and 5–95% percentile ranges of the MPI-ESM1-2-LR ensemble members are shown by brown shading. A 25° longitude running mean is applied to highlight broader regional patterns. Trends that are statistically significant at the 5% level (based on a two-*t*ailed *t*-test) are highlighted with thicker lines.

To better resolve the longitudinal structure of moisture transport toward Antarctica, Fig. 2 presents trends in vertically integrated moisture flux along the Antarctic Circle (60–70°S). This latitude band is chosen because it is projected to experience significant year-round trends in synoptic activity, and by encircling Antarctica, plays a key role in controlling the amount of moisture that can enter the Antarctic. During the short-term period (1980–2022), reanalysis data show that significant JJA trends are confined to sectors in Eastern Antarctica (~60°–100°E) and Western Antarctica (~220°–240°E). In DJF, significant trends are limited to narrow regions near ~50°E in Eastern Antarctica and ~270°E in Western Antarctica. These localized signals, which were not significant in Fig. 1, emerge here due to meridional and longitudinal averaging. This averaging enhances the signal-to-noise ratio by suppressing internal variability that strongly influences trend detection at regional scales.

In contrast, the externally forced trend in CMIP6 models exhibits reduced zonal variability in short-term trends and enhanced statistical significance, particularly during DJF when the intensification is markedly stronger. In JJA, modelled trends are notably weaker than those in reanalyses over Eastern Antarctica (60°–100°E) and across the Ross Sea and Western Antarctica (160°–260°E). During DJF, reanalyses show much more positive trends between 90°–220°E and more negative trends from 250°–280°E. To assess the potential role of natural variability in the model–reanalysis differences, we examine the spread among members of the MPI-ESM1-2-LR large ensemble, which serves as a gauge of internal variability^40^. The ensemble spread aligns with CMIP6 models and generally encompasses reanalysis trends. This suggests that discrepancies—aside from intense regional differences over East and West Antarctica and the Ross Sea—can be plausibly attributed to internal variability. Averaging over broader sectors (Supplementary Fig. 1) further reduces the gap between models and reanalyses, yielding consistent overlap across all seasons.

Over the long term (1980–2100), trends become statistically significant across all longitudes and in both seasons, with the strongest increases under the SSP5-8.5 scenario. Similar to patterns observed at lower latitudes (Fig. 1), the long-term trends exhibit three local minima, which do not form a perfect longitudinal wavenumber-3 pattern. When integrated over the entire Antarctic circle, the trends in vertically integrated synoptic moisture transport $([\overline{{v}^{\mathrm{'}}{q}^{\mathrm{'}}}])$ account for 24% of the total moisture transport $([\overline{vq}])$ trend in JJA and 93% in DJF for SSP5-8.5, compared with 58% and 73% for SSP2-4.5 (Table 1). These results highlight the dominant role of synoptic variability in shaping seasonal moisture transport trends, particularly during summer.

**Table 1:** Vertically integrated moisture vapour transport trends (1980–2100; kg m^−1^ s^−1^ yr^−1^) averaged across the Antarctic circle (60–70°S) in function of season and future projection scenario

Season	Scenario	Synoptic	Total	Ratio
JJA	SSP2-4.5	−0.00870	−0.0150	0.58
	SSP5-8.5	−0.0161	−0.0675	0.24
DJF	SSP2-4.5	−0.00856	−0.0118	0.73
	SSP5-8.5	−0.0146	−0.0157	0.93

Trends are reported for synoptic eddies fluxes $\left[\overline{v{\mathrm{'}}q{\mathrm{'}}}\right]$, total fluxes $\left[\overline{{vq}}\right]$, and their ratio.

### Vertical structure of synoptic moisture transport trends

**Fig. 3:**
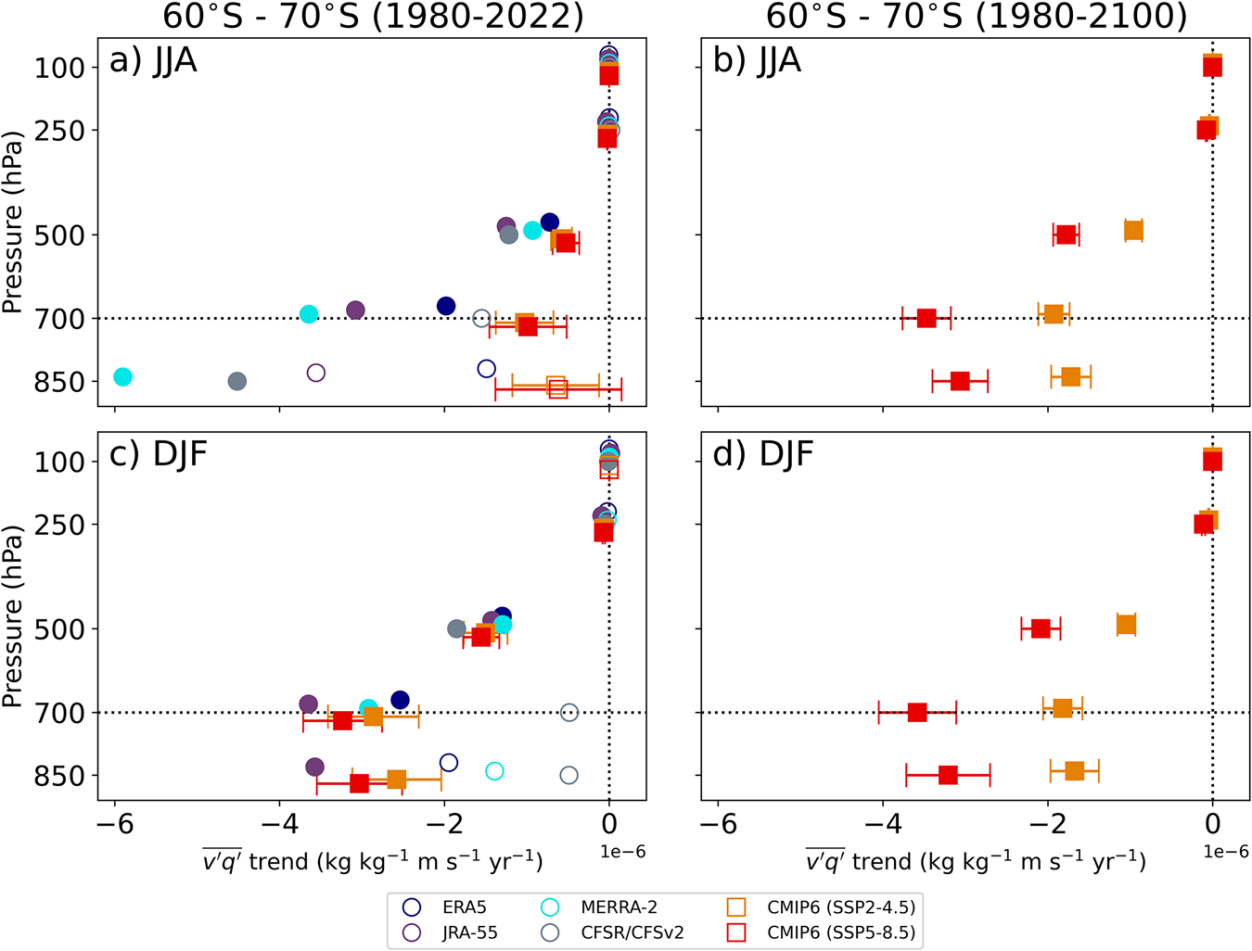
Vertical profile of synoptic moisture flux trends. Mean meridional moisture flux ($\overline{v{\mathrm{'}}q{\mathrm{'}}}$) trends averaged from 60°S to 70°S (kg kg^−1^ m s^−1^ yr^−1^) are contrasted between reanalyses (circles) and CMIP6 models (squares) for (left) short-term (1980–2022) and (right) long-term (1980–2100) CMIP6 projections. They are shown separately for (**a**, **b**) winter (JJA) and (**c**, **d**) summer (DJF). The 95% confidence interval on the CMIP6 model mean, as a measure of spread amongst models, are shown with error bars. Statistically significant trends for the reanalyses or model mean (*p* < 0.05) are shown with filled markers. The 700-hPa level is indicated with a dashed horizontal line.

Vertical profiles of zonal-mean meridional eddy moisture flux $(\overline{{v}^{\mathrm{'}}{q}^{\mathrm{'}}}$) trends are then shown (Fig. 3) for the same latitude band as Fig. 2 (60°S to 70°S). The trends are shown from 850 hPa and above since the levels below intersect too frequently with topography and ocean surface to allow for a robust analysis of trends. Trends are consistently negative, corresponding to the amplification of southward fluxes towards Antarctica. Except for MERRA-2, CFSR/CFSv2, and JRA-55 in JJA, the strongest signals are found at 700 hPa, above the climatological maximum that is located closer to the surface (Supplementary Fig. 2). We note that the climatological vertical profile of moisture fluxes is broadly consistent between CMIP6 model-mean and reanalyses, although the models tend to slightly overestimate transport near 500 hPa and underestimate it near 850 hPa.

Short-term (1980-2022) trends in CMIP6 models exhibit pronounced seasonal differences with a marked amplification in DJF, compared to JJA. In JJA, reanalyses exhibit greater trends compared to CMIP6 models, while in DJF, reanalysis trends are slightly smaller on average. The simulated zonal-mean meridional eddy moisture flux trends show limited sensitivity to emission scenarios (SSP2-4.5 *versus* SSP5-8.5). Trends are usually statistically significant at 700 and 500 hPa in reanalyses (except CFSR/CFSv2 at 700 hPa) and models, but less so at 850 hPa, likely due to reduced signal-to-noise ratio associated with internal variability in reanalyses. Averaging across CMIP6 models enhances the signal-to-noise ratio as it suppresses internal variability, improving trend detectability.

Unlike short-term trends in CMIP6 models, which show little sensitivity to emission scenarios, long-term trends (1980–2100) differ between SSP2-4.5 and SSP5-8.5, with the latter nearly twice as large as the former. All long-term trends are statistically significant in the lower troposphere, indicating robust increases in synoptic moisture transport under sustained warming.

### Temporal evolution of moisture transport

**Fig. 4:**
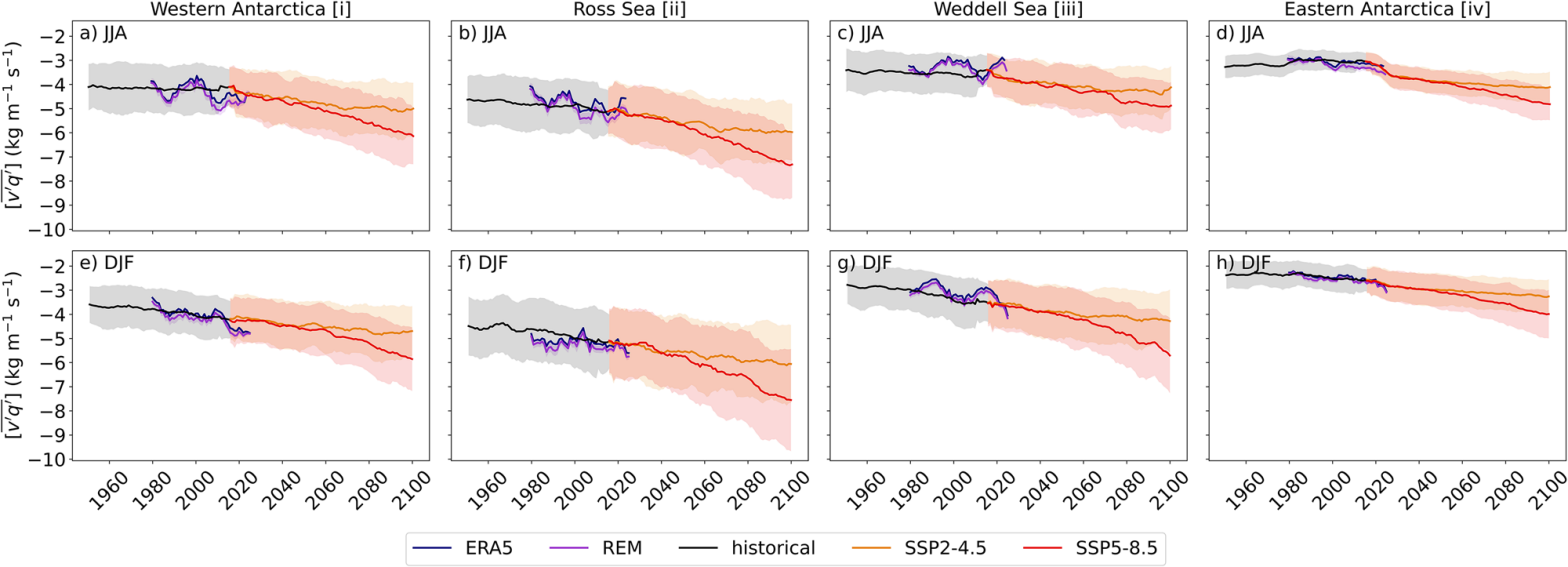
Time series of moisture transport across the Antarctic circle. Time series of vertically integrated synoptic meridional moisture fluxes ($\left[\overline{v\mathrm{'}q\mathrm{'}}\right]$; kg m^−1^ s^−1^) averaged over four representative sectors encircling Antarctica (columns; sectors illustrated in Fig. 1) in (**a**–**d**) JJA and (**e**–**h**) DJF in reanalyses (ERA5 and the reanalysis ensemble mean [REM], the mean of the four reanalyses considered in this study [ERA5, JRA-55, MERRA-2, and the merger of CFSR and CFSv2]) and CMIP6 models. Solid lines illustrate the REM and CMIP6 model ensemble averages, and the shading illustrates an $\pm 1{\rm{\sigma }}$ interval among ensemble members.

To illustrate the nonlinear progression of moisture transport under global warming, Fig. 4 presents the temporal evolution of meridional moisture fluxes for four representative sectors encircling Antarctica at 60–70°S. Spatial averaging over these broader regions further enhances the signal-to-noise ratio, allowing regional changes to emerge more clearly. All four sectors are projected to experience negative trends, indicating amplified synoptic-scale meridional moisture transport. The greatest changes are projected over the Ross Sea sector, while comparatively weaker trends are projected over Eastern Antarctica. Notably, the Eastern Antarctica sector spans a broad area encompassing both weak and strong regional signals, which average out to produce more moderate sector-wide trends (Figs. 1 and 2). Despite this moderation, the trend remains statistically significant due to the improved signal-to-noise ratio achieved through spatial averaging.

The sensitivity of long-term changes in moisture fluxes to CO_2_ emissions is also clear in Fig. 4 where the amplification of moisture fluxes accelerates continuously over time in SSP5-8.5 but flattens past ~2070 in SSP2-4.5. It is worth mentioning that Eastern Antarctica is affected by a weak positive trend (reduction) in the early historical period, which is consistent with the absence of a warming trend in the region during the same period^41^.

### Influence of internal variability

**Fig. 5:**
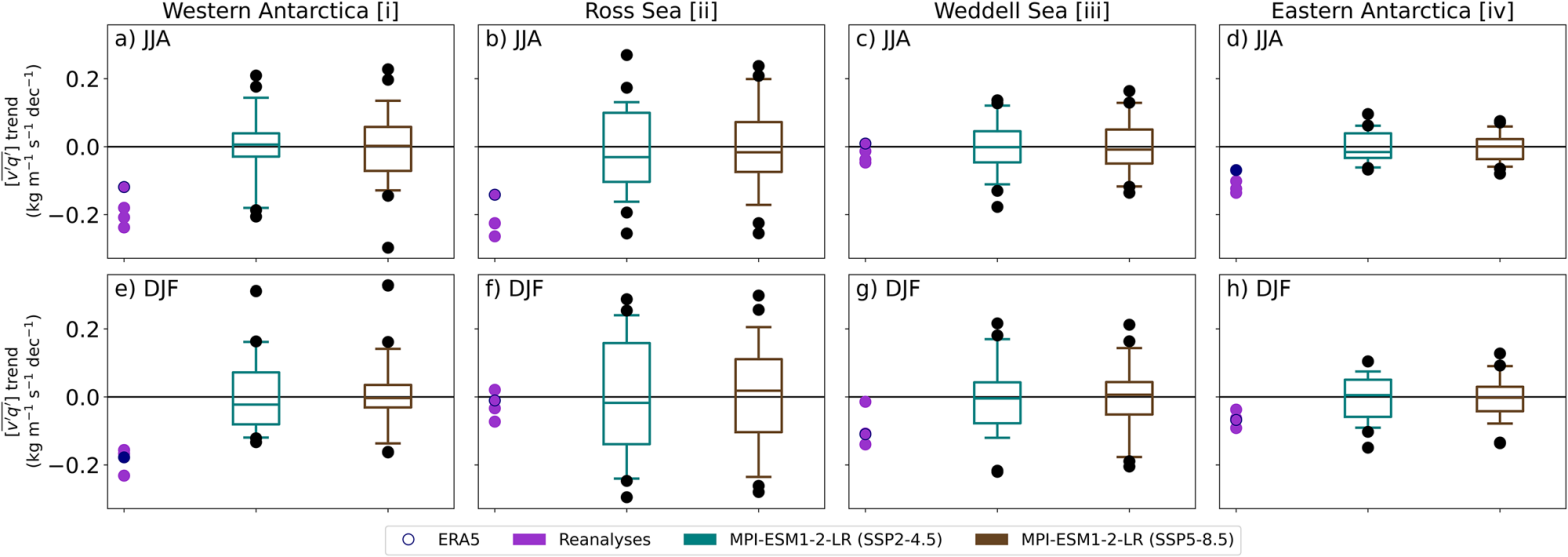
Emergence of trends. Meridional moisture flux trends (1980-2022; $[\overline{v\mathrm{'}q\mathrm{'}}]$; kg m^-1^ s^-1^ dec^-1^) averaged over four representative sectors encircling Antarctica (columns; sectors illustrated in Fig. 1) in (**a**–**d**) JJA and (**e**–**h**) DJF are contrasted between ERA5, other reanalyses, and the MPI-ESM1-2-LR large ensemble from which externally-forced climate variability (the ensemble mean) is removed before assessing trends. The inter-quartile range and median of the large ensemble are shown with boxes, and the 5-95 percentile range is shown with whiskers. Outliers are shown with black circles.

Next, we compare trends in reanalyses with those in the MPI-ESM1-2-LR ensemble members after removing the ensemble mean, thereby isolating internal variability (Fig. 5). We find that only in Eastern Antarctica [iv] during JJA and in Western Antarctica [i] during DJF do trends in ERA5 and other reanalyses fall below the 5th percentile of ensemble members. This suggests that the externally forced component of observed trends has emerged from internal variability only in these specific regions and seasons. For the remaining areas, trends in reanalyses can be plausibly attributed to natural climate variability.

### Drivers of moisture flux amplification

**Fig. 6:**
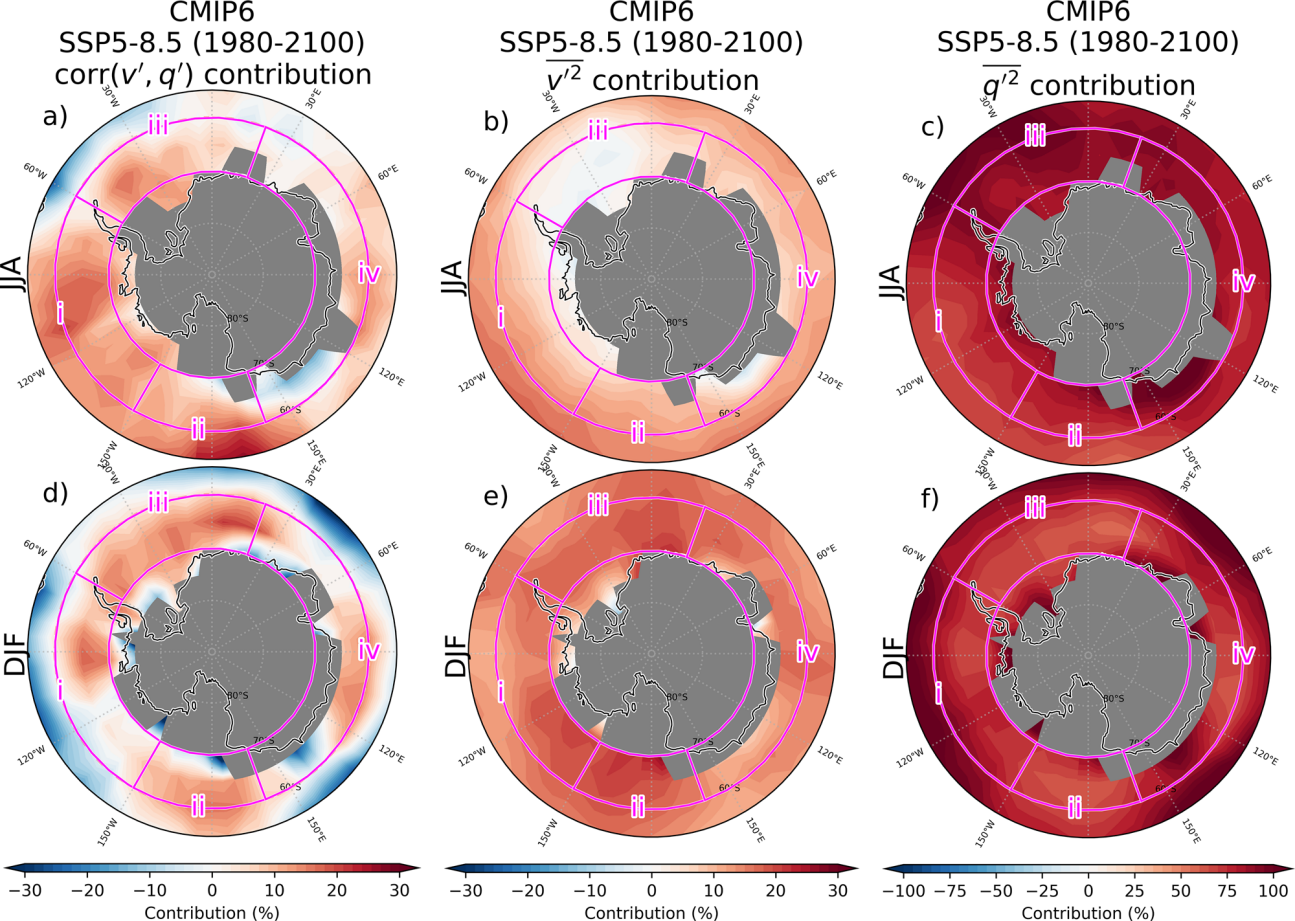
Contribution of different properties of synoptic eddies to moisture transport trends. The relative contributions of each RHS term of Eq. (2) to $\overline{v\mathrm{'}q\mathrm{'}}$ trends (1980-2100; SSP5-8.5 scenario) at 700 hPa are shown in (**a**–**c**) JJA and (**d**–**f**) DJF for (left; **a**, **d**) 1st RHS term, the efficiency contribution, (middle; **b**, **e**) 2nd RHS term, the wind contribution, and (right; **c**, **f**) 3rd RHS term, the moisture contribution. The contribution is shown here as the percentage of $\Delta \overline{{{{v}}}{\mathrm{'}}{{{q}}}{\mathrm{'}}}/\triangle t$. Contributions are assessed through linear regression. Areas where the 700 hPa level falls under the topography and where the ratios cannot be calculated due to limitations in numerical resolution (when the denominators are too small) are masked in grey. Note that the colour scale differs between the first two columns and the last column.

The projected amplification of moisture fluxes in CMIP6 models may result from changes in the amplitude of wind anomalies, moisture anomalies, or the correlation between both, which serves as a measure of the efficiency of synoptic systems in transporting moisture (see Eqs. (1) and (2) in methods for such decomposition). To examine their respective contributions, each term of Eq. (2) is illustrated in Fig. 6. It is evident that moisture flux trends are primarily driven by the greater eddy moisture anomalies. This contribution is particularly large north of 60°S and decreases slightly over the 60–70°S band, where the contribution of the correlation between $v\mbox{'}$ and $q\mbox{'}$ increases slightly. This suggests that the configuration of synoptic eddies becomes moderately more favourable for the transport of moisture towards Antarctica.

In summer, a wavenumber-3 pattern is observed in the contribution from trends in the correlation between $v\mathrm{'}$ and $q\mathrm{'}$ (first right-hand side term of Eq. (2); Fig. 6d). This pattern shows that sectors of enhanced trends (Figs. 1d and 2) also experience an increase in the efficiency of moisture fluxes. In addition, we identify a wavenumber-3 pattern in ${corr}\left({v}\mathrm{'},{q}\mathrm{'}\right)$ trends as well as a clear wavenumber-3 component in the amplification of $\sqrt{\overline{{v\mathrm{'} }^{2}}}$ (Supplementary Fig. 3) and in moisture anomaly trends $\left(\sqrt{\overline{{q\mathrm{'} }^{2}}}\right)$. The climatological wavenumber 3 patterns observed in these fields (not shown) correspond to stationary waves^37^.

It is important to note that the net contribution of each of these components to moisture flux trends, as derived from the decomposition in Eq. (2), results from their respective trends multiplied by the climatological properties of the other terms, which also exhibit zonal asymmetries. Therefore, the wavenumber-3 pattern in meridional moisture flux trends cannot be attributed to a single factor alone. Supplementary Fig. 4 displays the net contribution to these asymmetries. All three terms contribute wavenumber-3 anomalies to varying degrees.

A similar decomposition was also applied to ERA5 trends (not shown), but the interpretation proved challenging due to the spatially inhomogeneous trends in moisture fluxes likely driven by the strong presence of internal variability in the record, leading to a low signal-to-noise ratio.

**Fig. 7:**
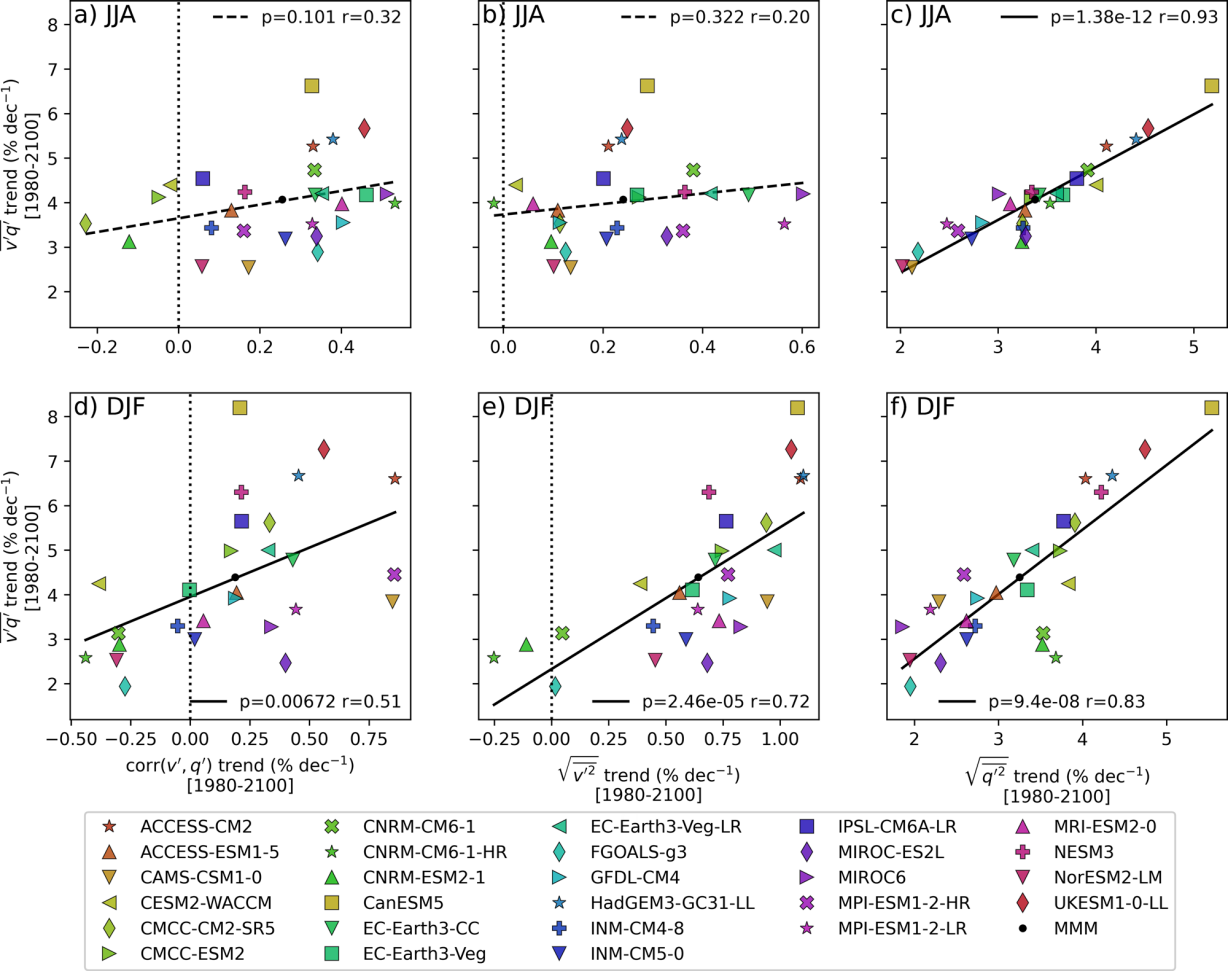
Sources of inter-model spread. Relationship between moisture flux trends (*y*-axes;) and trends in the three components (columns) of the mean meridional moisture fluxes (Eq. 1) (*x*-axes) amongst the CMIP6 models at 700 hPa across the latitude band of the Antarctic circle 60°–70°S for the SSP5-8.5 scenario (1980-2100) in (**a**–**c**) JJA and (**d**–**f**) DJF. Trends are shown relative to the climatological values over 1980–2022, expressed as a percentage. The *p*-value and correlation on the linear fit amongst CMIP6 models are indicated in each panel. Significant trends are shown with solid lines (*p* < 0.05).

The role of these components of moisture flux transport across the Antarctic Circle in contributing to inter-model uncertainties is further investigated (Fig. 7). Models that tend to exhibit comparatively larger DJF trends of meridional moisture fluxes ($\overline{v\mathrm{'}q\mathrm{'} }$) also tend to exhibit enhanced trends in meridional wind anomalies $(\sqrt{\overline{{v\mathrm{'}}^{2}}})$, specific humidity anomalies $(\sqrt{\overline{{q\mathrm{'}}^{2}}})$, and correlation between meridional wind anomalies and specific humidity anomalies. In JJA, only $\sqrt{\overline{{q\mathrm{'}}^{2}}}$ trends are significantly correlated with $\overline{v\mathrm{'}q\mathrm{'} }$ trends. This is explained in part by a decoupling between the typical magnitude of wind and moisture anomalies in JJA compared to DJF (Supplementary Fig. 5). This decoupling may be partly attributed to the presence of sea ice during winter, which limits the moisture supply to the atmosphere by restricting ocean–atmosphere exchange. Although trends in $\sqrt{\overline{{v\mathrm{'}}^{2}}}$ are more strongly correlated with trends in $\overline{v\mbox{'}q\mbox{'} }$ during DJF, its relative amplification compared to the climatological mean is small, resulting in only a modest contribution to the net amplification of meridional moisture fluxes. Notably, the relationship appears nonlinear with disproportionately large increases in $\overline{v\mathrm{'}q\mathrm{'} }$ when $\sqrt{\overline{{v\mathrm{'}}^{2}}}$ is high. Similar, albeit more noisy, inter-model relationships are found for short-term trends (Supplementary Fig. 6). For this period, the linear fits with $v\mbox{'}$ - $q\mbox{'}$ correlation and meridional winds are significant throughout the year. Reanalyses with smaller trends in $\sqrt{\overline{{q\mathrm{'}}^{2}}}$ (e.g., ERA5) also exhibit smaller trends in $\overline{v\mbox{'}q\mbox{'} }$, consistent with the behaviour observed in CMIP6 models.

**Fig. 8:**
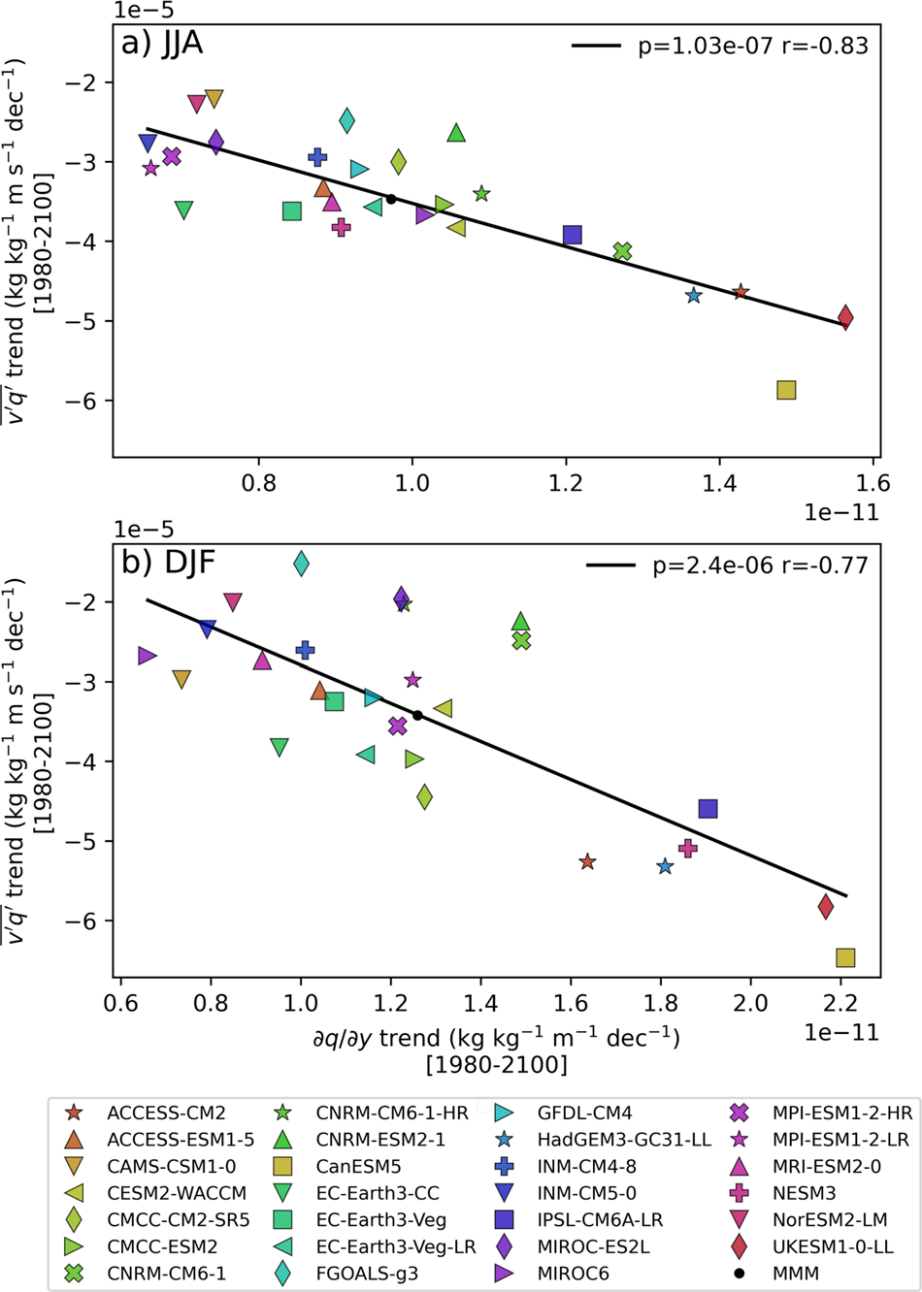
Meridional moisture gradient as a large-scale constraint on synoptic-scale moisture fluxes. As in Fig. 7, but showing the relationship between trends in synoptic moisture transport and trends in the background meridional moisture gradient in (**a**) JJA and (**b**) DJF. Note that, unlike Fig. 7, raw (unnormalized) trends are shown.

As an alternative perspective, we examine the relationship between trends in moisture transport and trends in the background meridional moisture gradient. Because lower latitudes moisten more rapidly than Antarctica under Clausius-Clapeyron scaling, synoptic eddies can act to relax the amplified gradient by enhancing poleward moisture transport. This diffusive interpretation is also evaluated across the multi-model ensemble (Fig. 8). In both seasons, we find a significant cross-model connection between trends in the moisture gradient and trends in synoptic moisture transport, with correlation coefficients of −0.83 in winter and −0.77 in summer.

Overall, these findings suggest that better constraining atmospheric moisture content is essential for reducing uncertainties in moisture transport projections.

### Eddy structural changes under future warming

**Fig. 9:**
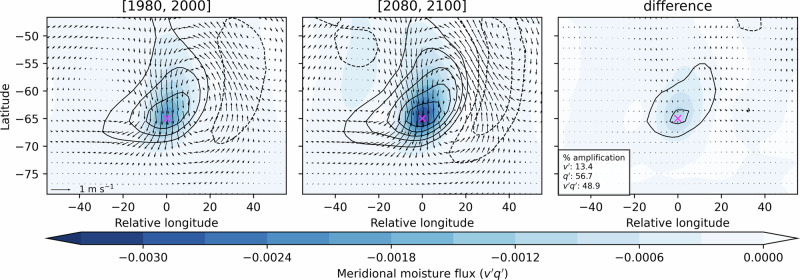
Changing eddy structure and amplitude. The structure of wind (arrows), meridional moisture flux (shading), and specific humidity (black contours) obtained with one-point correlation analysis (see methods) at 700 hPa in DJF is shown for (left) 1980–2000 and (middle) 2080–2100 in SSP5-8.5. The difference between the two periods is shown to the right. The reference latitude-longitude grid point is shown with a magenta x in each panel. The percentage of amplification of each component at the reference grid point is indicated in the legend.

Changes in eddy structure contributing to moisture flux trends are assessed using one-point regression analysis (Fig. 9; see Methods). Across all periods analyzed, enhanced synoptic-scale poleward moisture transport is consistently associated with cyclonic circulation anomalies to the west and anticyclonic circulation anomalies to the east – which is consistent with the location of atmospheric rivers with respect to cyclonic and anticyclonic systems^23^ and anomalous circulation patterns associated with temperature extremes^24,42^. While the spatial structure of these synoptic circulation features remains stable between 1980 and 2000 and 2080 and 2100, the amplitude of eddies increases substantially. Wind anomalies strengthen modestly (13.4%), while specific humidity anomalies and meridional moisture flux anomalies intensify more sharply (56.7% and 48.9%, respectively), underscoring the dominant role of moisture perturbations in influencing future synoptic eddies.

## Discussion

This study provides robust evidence that synoptic-scale moisture transport to Antarctica is undergoing significant changes under global warming. While short-term trends show limited scenario sensitivity, long-term projections reveal substantial amplification—particularly under high-emission scenarios. These changes are most pronounced in summer and are driven primarily by an increase in moisture content rather than circulation changes.

While a large amplification of moisture transport to Antarctica is seen in some mid and high-latitude sectors of the reanalysis record, these trends are rarely significant along the Antarctic coast, consistent with findings from previous studies^8,29^. In contrast, the externally-forced trends captured by the CMIP6 ensemble mean simulate widespread amplifying fluxes along the Antarctic coast, particularly in summer for the same historical period. The MPI-ESM1-2-LR ensemble spread shows that internal variability potentially explains the observed differences, though model bias remains a possibility. Nonetheless, reanalysis trends in Western Antarctica, the Ross Sea, and Eastern Antarctica are the exception, as their amplification clearly exceeds the range of internal variability. Differences may also arise from artificial trends stemming from changes in observing systems^43^, particularly in data-sparse regions like Antarctica, where reanalyses heavily rely on satellite observations. Our results also indicate that reanalysis-derived trends have not yet emerged as statistically distinct from the range of plausible internal variability across the entire Antarctic Circle.

Future projections from CMIP6 models reveal a statistically robust and substantial amplification of moisture transport across the Antarctic Circle by synoptic eddies. This amplification is highly sensitive to the emission scenarios, with accelerating trends under SSP5-8.5 and plateauing trends under SSP2-4.5. It is the largest at around 180°E, north of the Ross Sea, potentially influencing Antarctic bottom water formation^44^. These trends in synoptic moisture transport account for a substantial fraction of the total moisture transport trends, ranging from 24–58% in winter and 73–93% in summer, depending on the emission scenario.

Projected trends are primarily driven by increases in the moisture content of synoptic eddies, a thermodynamic response to warming^34,35^, which also emerges as the dominant source of uncertainty across model projections. This suggests that future trends in moisture transport towards Antarctica are not primarily driven by changes in the kinematic properties of storm activity, as assessed with synoptic-scale wind variability. This distinction is particularly evident in winter when the intensification of the storm track leads to only modest changes in synoptic-scale meridional wind variability, with limited correlation to moisture transport trends amongst CMIP6 models. Rather, trends in moisture transport are found to be strongly correlated with the trends in background meridional moisture gradient, suggesting that when this gradient intensifies, synoptic eddies act to relax this gradient by enhancing poleward moisture transport. We note that the 2.5° × 2.5° grid used in this study does not fully resolve sub‑grid processes such as frontal structures, which may influence the background meridional moisture gradient.

The amount of moisture reaching Antarctica is also strongly related to the ambient temperatures along the Antarctic Circle, especially in winter (Supplementary Fig. 7). This relationship can be explained by the Clausius-Clapeyron relationship whereby warming increases atmospheric moisture holding capacity. As a result, warmer saturated poleward air streams carry significantly more moisture than their colder, unsaturated equatorward counterparts, amplifying synoptic-scale variability in specific humidity and moisture fluxes. Reducing uncertainties in future projections of moisture transport to Antarctica may therefore depend on improved constraints on atmospheric temperature over the Antarctic Circle and its coastal regions.

## Methods

### Data

Reanalysis data are obtained for the fifth-generation reanalysis produced at ECMWF (ERA5^45^), the Japanese 55-year Reanalysis (JRA-55^46^), the second Modern-Era Retrospective analysis for Research and Applications (MERRA-2^47^), the Climate Forecast System Reanalysis (CFSR^48^), and the Climate Forecast System Version 2 (CFSv2^49^). CFSR, whose production stopped in 2011, and CFSv2, its replacement, are merged to form a single dataset. A reanalysis ensemble mean (REM) is obtained by averaging these four reanalyses. Results based on ERA5 are emphasized because it has been suggested as the most appropriate dataset for assessing moisture in Antarctica^17^.

The historical, Shared Socioeconomic Pathway 2-4.5 (SSP2-4.5), and Shared Socioeconomic Pathway 5-8.5 (SSP5-8.5) scenarios are obtained for 27 models participating in the sixth phase of the Coupled Model Intercomparison Project^36^ (CMIP6): ACCESS-CM2, ACCESS-ESM1-5, CAMS-CSM1-0, CESM2-WACCM, CMCC-CM2-SR5, CMCC-ESM2, CNRM-CM6-1, CNRM-CM6-1-HR, CNRM-ESM2-1, CanESM5, EC-Earth3-CC, EC-Earth3-Veg, EC-Earth3-Veg-LR, FGOALS-g3, GFDL-CM4, HadGEM3-GC31-LL, INM-CM4-8, INM-CM5-0, IPSL-CM6A-LR, MIROC-ES2L, MIROC6, MPI-ESM1-2-HR, MPI-ESM1-2-LR, MRI-ESM2-0, NESM3, NorESM2-LM, and UKESM1-0-LL. The SSP2-4.5 “Middle of the Road” scenario corresponds to a world that continues along a trajectory where social, economic, and technological trends remain consistent with historical patterns, and the SSP5-8.5 “Fossil-fuelled Development” scenario assumes an economy that is based on an intense demand for fossil-fuel-based energy^50,51^. Only the first ensemble member of these models is considered, except for the MPI-ESM1-2-LR large ensemble, for which 30 ensemble members are used to robustly assess the impact of internal climatic variability. Unlike the spread among CMIP6 models, which arises from both internal variability and differences in model physics, the spread among members of the large ensemble is primarily driven by internal variability. This model is used here because it is the only CMIP6 large ensemble that provides a sufficiently large number of members with available output of required variables at a temporal resolution suitable for analyzing synoptic moisture fluxes. When analyzing trends, the historical and SSP scenarios are combined to make a seamless record.

Variables considered include the meridional wind velocity ($v$; m s^−1^), zonal wind velocity ($u$; m s^−1^), specific humidity ($q$; kg kg^−1^), and temperature (*T*: K). All datasets are interpolated to the same 2.5 by 2.5 degrees latitude and longitude grid and a subset of common pressure levels are kept (100, 250, 500, 700, 850, and 1000 hPa) before calculations due to the limited of vertical levels provided for CMIP6 models.

### Moisture fluxes and decomposition

Synoptic meridional moisture fluxes $({{v}\mathrm{'}q\mathrm{'} })$ are calculated based on daily-averaged $v$ and $q$ onto which a 10-day high-pass filter is applied (denoted with primes). This filtering primarily retains synoptic-scale weather systems (see Fig. 9). Seasonal-mean fluxes (denoted with overbars), based on the definition of the Pearson correlation coefficient (${corr}$), can be expressed as

1 $$\overline{{v}{\mathrm{'}}{q}{\mathrm{'}}}={corr}\left({v}\mathrm{'},{q}\mathrm{'}\right)\sqrt{\overline{{{v}{{\mathrm{'}}}}^{2}}}\sqrt{\overline{{{q}{{\mathrm{'}}}}^{2}}}$$

The contribution of each term to trends of $\overline{{v}\mathrm{'}q\mathrm{'} }$ is estimated based on the product rule of derivation as follows

2 $$\frac{\Delta \overline{{{v}\mathrm{'}q}\mathrm{'}}}{\Delta t}=\mathop{\underbrace{\frac{\Delta {corr}\left({v}\mathrm{'},{q}\mathrm{'}\right)}{\Delta t}\left\langle \sqrt{\overline{{v\mathrm{'} }^{2}}}\right\rangle \left\langle \sqrt{\overline{{q\mathrm{'} }^{2}}}\right\rangle }}\limits_{\mathrm{efficiency\; contribution}}+\mathop{\underbrace{\left\langle {corr}\left({v}\mathrm{'},{q}\mathrm{'}\right)\right\rangle \frac{\Delta \sqrt{\overline{{v\mathrm{'} }^{2}}}}{\Delta t}\left\langle \sqrt{\overline{{q\mathrm{'} }^{2}}}\right\rangle }}\limits_{\mathrm{wind\; contribution}}+\mathop{\underbrace{\left\langle {corr}\left({v}\mathrm{'},{q}\mathrm{'}\right)\right\rangle \left\langle \sqrt{\overline{{v\mathrm{'} }^{2}}}\right\rangle \frac{\Delta \sqrt{\overline{{q\mathrm{'} }^{2}}}}{\Delta t}}}\limits_{\mathrm{moisture\; contribution}}$$

where $\triangle X/\triangle t$ is assessed here by linearly regressing $X$ with respect to time (t) and where $\left\langle X\right\rangle$ represents the climatological mean.

When moisture fluxes are shown as vertically integrated fluxes (denoted by square brackets) they are obtained by integrating fluxes from the surface to 100 hPa:

3 $$[\overline{v\mathrm{'} q\mathrm{'} }]=-\frac{1}{g}{\int }_{{ps}}^{100}\overline{v\mathrm{'}q\mathrm{'} }{dp}$$

where ${ps}$ is the surface pressure and *g* is the acceleration due to gravity (9.81 m s^−2^).

It is worth noting that, when vertically integrated moisture fluxes are seasonally averaged over a circular domain enclosing Antarctica, as done in this study, and given that evaporation over Antarctica is negligible, the resulting flux represents the total amount of water vapour that ultimately precipitates within the enclosed domain.

We further note that the high-pass filtering employed to extract synoptic variability is incompatible with missing data. Therefore, $\overline{v\mathrm{'} q\mathrm{'} }$ is unavailable for a given pressure level when this level is located below the surface for one day within a season. The method is therefore not adequate to measure near-surface fluxes. It should be adequate, however, to assess the eddy poleward transport of moisture, which happens primarily above 900 hPa surrounding Antarctica^29^. To ensure a fair comparison of vertically-integrated moisture fluxes, a common land mask is applied amongst reanalyses and models before calculating the vertical integral. We found however, that the results are not sensitive to whether a common land mask is used or not.

For the REM and the CMIP6 multi-model mean, moisture fluxes are first averaged seasonally and then across datasets, after which trends are assessed from the resulting mean time series.

### Eddy structure analysis

Changes in eddy structure between the periods 1980–2000 and 2080–2100 are assessed by performing a one-point regression analysis^52^ at 700 hPa and 65°S. The reference variable is the daily synoptic meridional moisture flux, $v\mathrm{'}q\mathrm{'}$. A reference longitude $\lambda$ (e.g., 60 ° W) is first selected, defining a reference point at 60°W, 65°S. The $v\mathrm{'}q\mathrm{'}$ time series at this point is standardized separately for each period. This yields a unit-variance reference series used to regress the spatial fields, allowing direct comparison of eddy structures across the two periods.

The structure of eddies associated with typical fluctuations of $v\mathrm{'}q\mathrm{'}$ at the reference point is obtained by regressing atmospheric fields at each surrounding grid point onto the standardized reference time series. Regression maps are generated over a domain spanning 45° S to 80° S meridionally 50° west to 50° east of the reference longitude (e.g., from 10°W - 110°W in this example). The regressed variables include specific humidity ($q\mathrm{'}$), meridional ($v\mathrm{'}$) and zonal ($u\mathrm{'}$) wind components, as well as meridional moisture flux ($v\mathrm{'}q\mathrm{'}$) itself.

This procedure yields spatial patterns that represent the eddy structures associated with fluctuations in $v{\mathrm{'}}q{\mathrm{'}}$ at the reference location, equivalent to to a standard one-point regression analysis centered at 60° W, 65° S. To derive a pattern representative of multiple longitudes—for example, 60° W and 100° W—the regression framework is extended by first combining information from both reference points before performing the regression.

Specifically, the regression domain surrounding each reference longitude is recast into a coordinate system defined relative to its respective reference point. The standardized ${v}^{{\prime} }{q}^{{\prime} }$ time series from the two reference locations are then concatenated, effectively pooling the associated samples into a single composite reference series. Atmospheric variables expressed in the same relative coordinate system are subsequently regressed onto this combined reference time series. The resulting regression map thus captures the eddy structure associated with the combined variability of ${v}^{{\prime} }{q}^{{\prime} }$ from both the 60° W and 100° W reference longitudes.

To obtain an eddy structure that is representative of conditions around the entire Antarctic Circle, we apply the same procedure to the regression domains centered at all longitudes along 65°S. This regression analysis is performed for DJF using CESM2-WACCM, whose meridional moisture flux trends closely resemble the multi-model mean (Fig. 7).

### Field significance

To assess the robustness of spatial patterns in trend significance, we evaluated field significance^53^, which accounts for multiple hypothesis testing and spatial correlation among grid points. We used a false discovery control value (αFDR) of 0.1.

## Supplementary information

Supplementary information

## Data Availability

MERRA-2 was obtained from the NASA Goddard Earth Sciences Data and Information Services Center (https://disc.gsfc.nasa.gov/datasets?project=MERRA-2). CFSR, CFSv2, and JRA-55 data were obtained from the research data archive (RDA; https://rda.ucar.edu/). ERA5 was obtained from the climate data store (10.24381/cds.bd0915c6). CMIP6 model data were obtained from the Earth System Grid Federation (e.g. https://esgf-node.llnl.gov/projects/cmip6/). Code can be provided upon request.

## References

[1] Wang, H. et al. Influence of sea-ice anomalies on Antarctic precipitation using source attribution in the Community Earth System Model. *Cryosphere* **14**, 429–444 (2020).

[2] Hanna, E. et al. Mass balance of the ice sheets and glaciers – Progress since AR5 and challenges. *Earth-Sci. Rev.* **201**, 102976 (2020).

[3] Rendfrey, T. S., Pettersen, C., Bassis, J. N. & Mateling, M. E. CloudSat observations show enhanced moisture transport events increase snowfall rate and frequency over Antarctic ice sheet basins. *J. Geophys. Res.: Atmos.* **129**, e2023JD040556 (2024).

[4] Budd, W. F., Reid, P. A. & Minty, L. J. Antarctic moisture flux and net accumulation from global atmospheric analyses. *Ann. Glaciol.* **21**, 149–156 (1995).

[5] Delaygue, G., Masson, V. & Jouzel, J. Climatic stability of the geographic origin of Antarctic precipitation simulated by an atmospheric general circulation model. *Ann. Glaciol.* **29**, 45–48 (1999).

[6] Slonaker, R. L. & Van Woert, M. L. Atmospheric moisture transport across the Southern Ocean via satellite observations. *J. Geophys. Res. Atmos.* **104**, 9229–9249 (1999).

[7] Connolley, W. M. & King, J. C. Atmospheric water-vapour transport to Antarctica inferred from radiosonde data. *Q. J. R. Meteorol. Soc.* **119**, 325–342 (1993).

[8] Tsukernik, M. & Lynch, A. H. Atmospheric Meridional Moisture Flux over the Southern Ocean: A Story of the Amundsen Sea. *J. Clim.* **26**, 8055–8064 (2013).

[9] Trenberth, K. E. & Stepaniak, D. P. Covariability of components of poleward atmospheric energy transports on seasonal and interannual timescales. *J. Clim.* **16**, 3691–3705 (2003).

[10] Liu, Q., Li, T. & Zhou, W. Impacts of Multi-Timescale Circulations on Meridional Moisture Transport. *J. Clim.* **34**, 1–64 (2021).

[11] Tietäväinen, H. & Vihma, T. Atmospheric moisture budget over Antarctica and the Southern Ocean based on the ERA-40 reanalysis. *Int. J. Climatol.* **28**, 1977–1995 (2008).

[12] Turner, J., Marshall, G. J. & Lachlan-Cope, T. A. Analysis of synoptic-scale low pressure systems within the Antarctic Peninsula sector of the circumpolar trough. *Int. J. Climatol.* **18**, 253–280 (1998).

[13] Simmonds, I., Keay, K. & Lim, E. P. Synoptic activity in the seas around Antarctica. *Month. Weather Rev.* **131**, 272–288 (2003).

[14] Uotila, P., Pezza, A. B., Cassano, J. J., Keay, K. & Lynch, A. H. A comparison of low pressure system statistics derived from a high-resolution NWP output and three reanalysis products over the Southern Ocean. *J. Geophys. Res. Atmos.* **114**, 1–19 (2009).

[15] Irving, D., Simmonds, I. & Keay, K. Mesoscale cyclone activity over the ice-free Southern Ocean: 1999-2008. *J. Clim.* **23**, 5404–5420 (2010).

[16] Sinclair, V. A. & Dacre, H. F. Which extratropical cyclones contribute most to the transport of moisture in the Southern Hemisphere? *J. Geophys. Res.: Atmos.* **124**, 2525–2545 (2019).

[17] Naakka, T., Nygård, T. & Vihma, T. Air moisture climatology and related physical processes in the antarctic on the basis of ERA5 reanalysis. *J. Clim.* **34**, 4463–4480 (2021).

[18] Papritz, L. et al. The role of extratropical cyclones and fronts for Southern Ocean freshwater fluxes. *J. Clim.* **27**, 6205–6224 (2014).

[19] Maclennan, M. L., Lenaerts, J. T. M., Shields, C. & Wille, J. D. Contribution of Atmospheric Rivers to Antarctic Precipitation. *Geophys. Res. Lett.* **49**, 1–8 (2022). 36246739 10.1029/2022GL100585 PMC9539845

[20] Gorodetskaya, I. V. et al. The role of atmospheric rivers in anomalous snow accumulation in East Antarctica. *Geophys. Res. Lett.* **41**, 6199–6206 (2014).

[21] Gorodetskaya, I. V., Silva, T., Schmithüsen, H. & Hirasawa, N. Atmospheric River Signatures in Radiosonde Profiles and Reanalyses at the Dronning Maud Land Coast, East Antarctica. *Adv. Atmos. Sci.* **37**, 455–476 (2020).

[22] Maclennan, M. L. et al. Climatology and surface impacts of atmospheric rivers on West Antarctica. *Cryosphere* **17**, 865–881 (2023).

[23] Guo, Y., Shinoda, T., Guan, B., Waliser, D. E. & Chang, E. K. M. Statistical Relationship between Atmospheric Rivers and Extratropical Cyclones and Anticyclones. *J. Clim.* **33**, 7817–7834 (2020).

[24] Lu, H. et al. Extreme warm events in the South Orkney Islands, Southern Ocean: Compounding influence of atmospheric rivers and föhn conditions. *Q. J. R. Meteorol. Soc.* **149**, 3645–3668 (2023).

[25] Franzke, C. L. E. & Harnik, N. Long-term trends of the atmospheric circulation and moist static energy budget in the JRA-55 reanalysis. *J. Clim.* **36**, 2959–2984 (2023).

[26] Chemke, R., Ming, Y. & Yuval, J. The intensification of winter mid-latitude storm tracks in the Southern Hemisphere. *Nat. Clim. Change* **12**, 553–557 (2022).

[27] Chemke, R. & Polvani, L. M. Linking midlatitudes eddy heat flux trends and polar amplification. *npj Clim. Atmos. Sci.* **3**, 1–8 (2020).

[28] Cox, T., Donohoe, A., Armour, K. C., Frierson, D. M. W. & Roe, G. H. Trends in atmospheric heat transport since 1980. *J. Clim.* **37**, 1539–1550 (2024).

[29] Dufour, A., Charrondière, C. & Zolina, O. Moisture transport in observations and reanalyses as a proxy for snow accumulation in East Antarctica. *Cryosphere* **13**, 413–425 (2019).

[30] Chemke, R. The future poleward shift of Southern Hemisphere summer mid-latitude storm tracks stems from ocean coupling. *Nat. Commun.* **13**, 1730 (2022). 35365654 10.1038/s41467-022-29392-4 PMC8975866

[31] Chang, E. K. M., Guo, Y. & Xia, X. CMIP5 multimodel ensemble projection of storm track change under global warming. *J. Geophys. Res.: Atmos.* **117**, 1–19 (2012).

[32] Uotila, P., Lynch, A. H., Cassano, J. J. & Cullather, R. I. Changes in Antarctic net precipitation in the 21st century based on Intergovernmental Panel on Climate Change (IPCC) model scenarios. *J. Geophys. Res. Atmos.* **112**, 1–19 (2007).

[33] Krinner, G., Largeron, C., Ménégoz, M., Agosta, C. & Brutel-Vuilmet, C. Oceanic forcing of Antarctic climate change: a study using a stretched-grid atmospheric general circulation model. *J. Clim.* **27**, 5786–5800 (2014).

[34] Grieger, J., Leckebusch, G. C. & Ulbrich, U. Net precipitation of Antarctica: thermodynamical and dynamical parts of the climate change signal. *J. Clim.* **29**, 907–924 (2016).

[35] Dalaiden, Q., Goosse, H., Lenaerts, J. T. M., Cavitte, M. G. P. & Henderson, N. Future Antarctic snow accumulation trend is dominated by atmospheric synoptic-scale events. *Commun. Earth Environ.* **1**, 62 (2020).

[36] Eyring, V. et al. Overview of the Coupled Model Intercomparison Project Phase 6 (CMIP6) experimental design and organisation. *Geosci. Model Dev. Discuss.* **8**, 10539–10583 (2016).

[37] van Loon, H. & Jenne, R. L. The zonal harmonic standing waves in the southern hemisphere. *J. Geophys. Res.* **77**, 992–1003 (1972).

[38] Goyal, R., Jucker, M., Gupta, A. S. & England, M. H. A New Zonal Wave-3 Index for the Southern Hemisphere. *J. Clim.* **35**, 5137–5149 (2022).

[39] Goyal, R., Jucker, M., Sen Gupta, A., Hendon, H. H. & England, M. H. Zonal wave 3 pattern in the Southern Hemisphere generated by tropical convection. *Nat. Geosci.* **14**, 732–738 (2021).

[40] Jain, S. et al. Importance of internal variability for climate model assessment. *npj Clim. Atmos. Sci.* **6**, 68 (2023).

[41] Xin, M. et al. West-warming East-cooling trend over Antarctica reversed since early 21st century driven by large-scale circulation variation. *Environmental Research Letters* **18**, (2023).

[42] Turner, J. et al. Extreme temperatures in the Antarctic. *J. Clim.* **34**, 2653–2668 (2021).

[43] Bromwich, D. H., Nicolas, J. P. & Monaghan, A. J. An Assessment of Precipitation Changes over Antarctica and the Southern Ocean since 1989 in Contemporary Global Reanalyses*. *J. Clim.* **24**, 4189–4209 (2011).

[44] Orsi, A. H., Johnson, G. C. & Bullister, J. L. Circulation, mixing, and production of Antarctic Bottom Water. *Prog. Oceanogr.* **43**, 55–109 (1999).

[45] Hersbach, H. et al. The ERA5 global reanalysis. *Q. J. R. Meteorol. Soc.* **146**, 1999–2049 (2020).

[46] Kobayashi, S. et al. The JRA-55 Reanalysis: General Specifications and Basic Characteristics. *J. Meteorol. Soc. Jpn. Ser. II* **93**, 5–48 (2015).

[47] Gelaro, R. et al. The Modern-Era Retrospective Analysis for Research and Applications, Version 2 (MERRA-2). *J. Clim.* **30**, 5419–5454 (2017). 10.1175/JCLI-D-16-0758.1 PMC6999672 32020988

[48] Saha, S. et al. The NCEP climate forecast system reanalysis. *Bull. Am. Meteorol. Soc.* **91**, 1015–1058 (2010).

[49] Saha, S. et al. The NCEP Climate Forecast System Version 2. *J. Clim.* **27**, 2185–2208 (2014).

[50] Riahi, K. et al. The Shared Socioeconomic Pathways and their energy, land use, and greenhouse gas emissions implications: An overview. *Glob. Environ. Change* **42**, 153–168 (2017).

[51] O’Neill, B. C. et al. The Scenario Model Intercomparison Project (ScenarioMIP) for CMIP6. *Geosci. Model Dev.* **9**, 3461–3482 (2016).

[52] Wallace, J. M. & Gutzler, D. S. Teleconnections in the Geopotential Height Field during the Northern Hemisphere Winter. *Month. Weather Rev.* **109**, 784–812 (1981).

[53] Wilks Dan, S. “The Stippling Shows Statistically Significant Grid Points”: How Research Results are Routinely Overstated and Overinterpreted, and What to Do about It. *Bull. Am. Meteorol. Soc.* **97**, 2263–2273 (2016).

