# Supplementary material for: Projected and historical amplification of moisture fluxes towards Antarctica by synoptic eddies
Source: NPJ Clim Atmos Sci. 2026 Apr 2;9(1):127. doi: 10.1038/s41612-026-01376-x (PMC13236580; doi:10.1038/s41612-026-01376-x)
Supplement: Supplementary file 1 — Supplementary information [file 41612_2026_1376_MOESM1_ESM.pdf]

npj climate and atmospheric science

Supplementary information for

"Projected and Historical Amplification of Moisture Fluxes towards Antarctica by Synoptic Eddies"

Patrick Martineau[1], Hua Lu[2], and Thomas J. Bracegirdle[2]

1 Japan Agency for Marine-Earth Science and Technology, Yokohama, Japan.

2 British Antarctic Survey, Cambridge, United Kingdom.

Contents of this file: Supplementary Figures 1 to 7

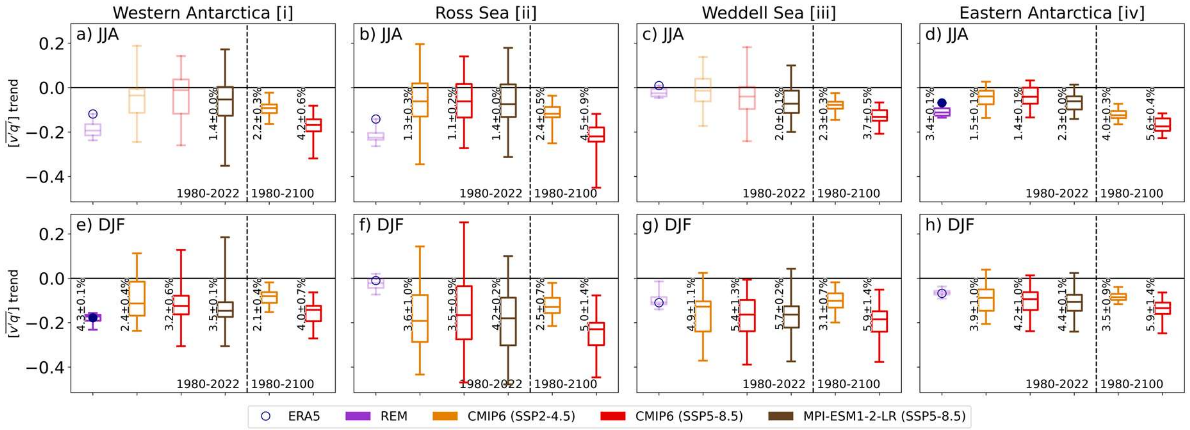

Supplementary Figure 1. Robustness of trends in models and reanalyses. Boxplot of vertically-integrated meridional moisture flux trends ($[\overline{v'q'}]$; kg m^{-1} s^{-1} dec^{-1}) extracted from reanalyses (ERA5 and all reanalyses included in the REM), CMIP6 model means for SSP2-4.5 and SSP5-8.5 scenarios, and the MPI-ESM1-2-LR large ensemble averaged over four representative sectors encircling Antarctica (columns; areas illustrated in Fig. 1) and for (a, b, c, d) winter and (e, f, g, h) summer. The median and inter-quartile ranges are shown with boxes and the full range is shown with whiskers. Trends are assessed for two periods (delimited by vertical dashed lines) whose limits are indicated at the bottom of each panel. The multi-reanalysis, multi-model, and multi-ensemble are illustrated with colors according to the legend. The percentage change relative to the climatology for the period 1980-2022 is indicated next to each box. Transparent boxes and whiskers indicate that trends of the multi-reanalysis or multi-model means are not significant at the 5% level according to a t-test.

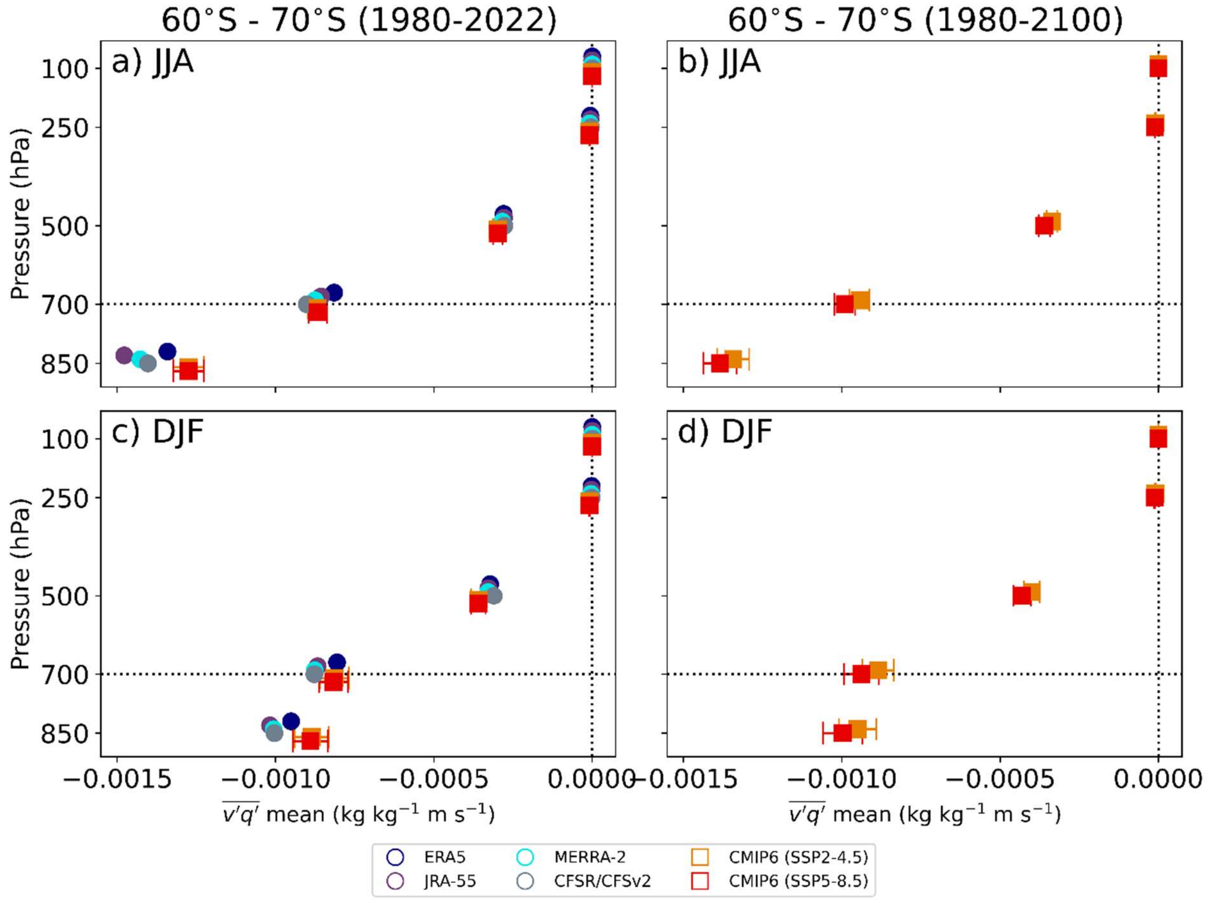

Supplementary Figure 2: Same as Fig. 3 but showing the mean meridional synoptic moisture flux climatology ($\overline{v'q'}$; kg kg^{-1} m s^{-1}) instead of its trend in (a, b) JJA and (c, d) DJF.

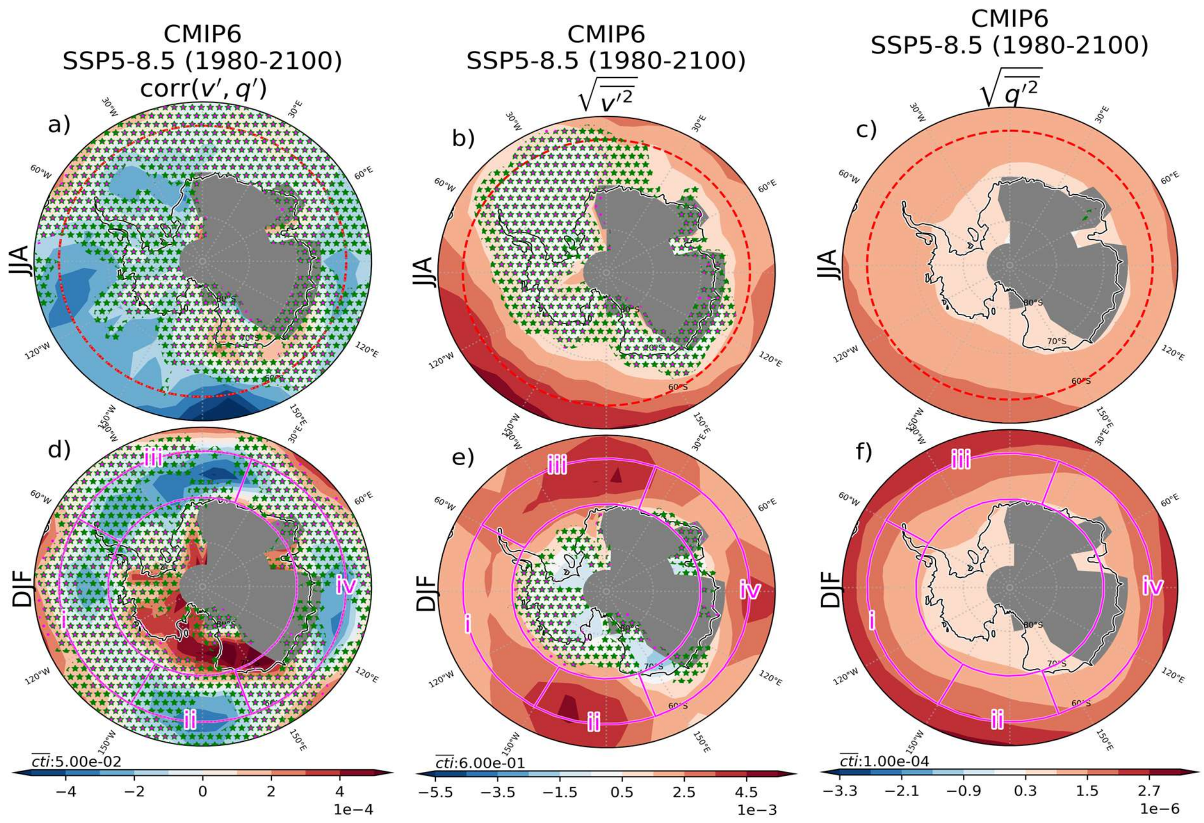

Supplementary Figure 3: Trends (1980-2100; SSP5-8.5 scenario) at 700 hPa are shown for (a, d) $corr(v', q')$, (b, e) $\sqrt{\overline{v'^2}}$, and (c, f) $\sqrt{\overline{q'^2}}$. Areas where the 700 hPa level falls under the topography are masked in grey.

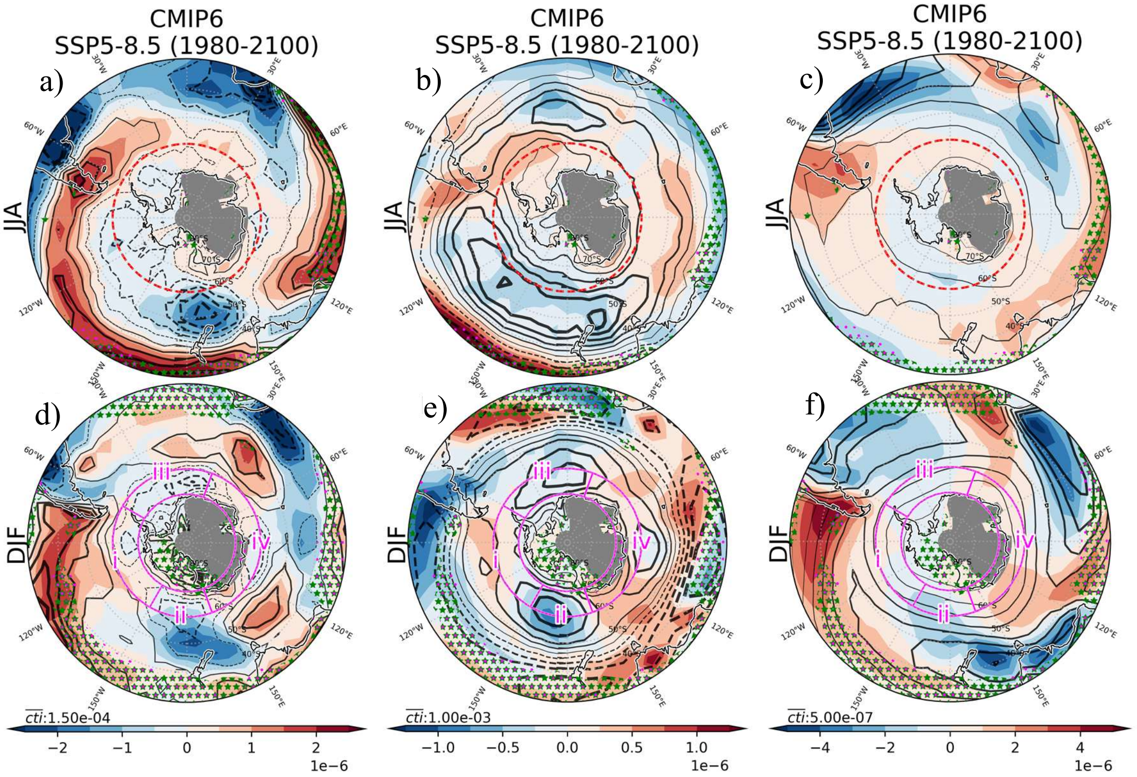

Supplementary Figure 4: Same as Fig. 6 except for the zonally asymmetric component of the net contribution (three terms of eq. (2); kg kg^{-1} m s^{-1} yr^{-1}) instead of their relative contribution in (a, b, c) JJA and (d, e, f) DJF. For reference, black contours show the trends of the following properties: $corr(v', q')$, $\sqrt{\overline{v'^2}}$, and $\sqrt{\overline{q'^2}}$) - already shown in Supplementary Fig. 3.

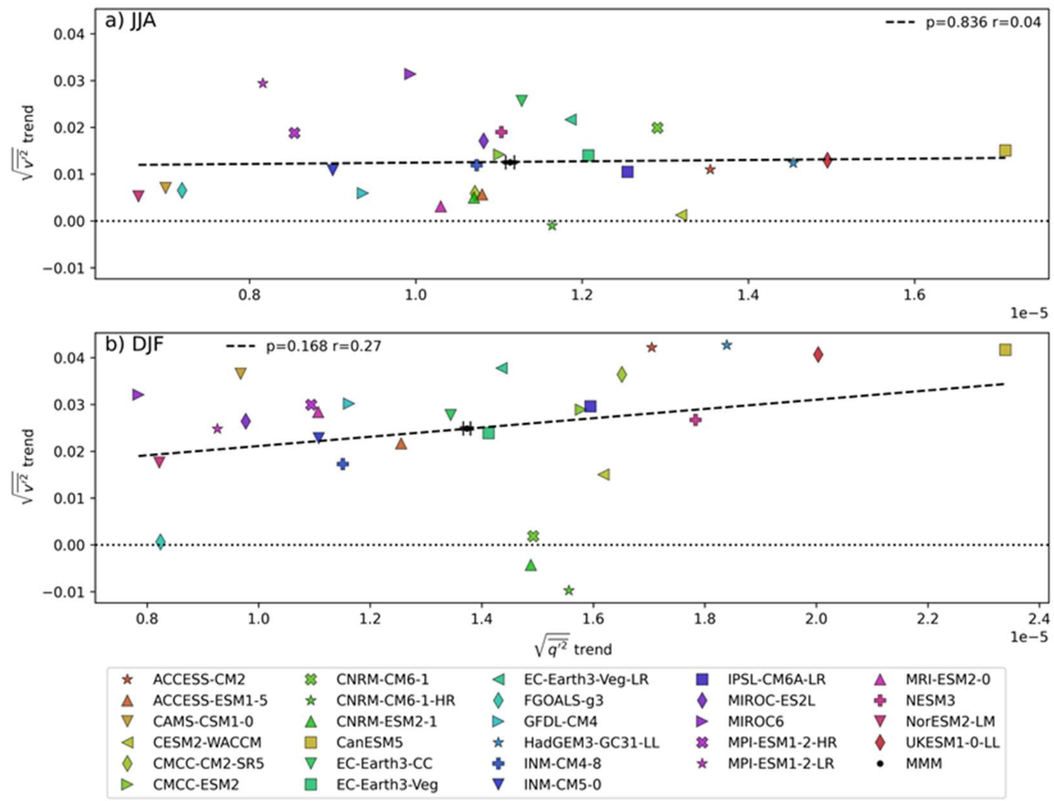

Supplementary Figure 5: Relationship between trends in the typical synoptic meridional wind magnitudes (*y*-axes; m s^{-1}) and trends in the typical synoptic specific humidity magnitudes (*x*-axes; kg kg^{-1}) amongst the CMIP6 models at 700 hPa over the Antarctic circle 60°-70°S for the SSP5-8.5 scenario (1980-2100) in (a) JJA and (b) DJF. The *p*-value and correlation amongst models on the trend are indicated in each panel. Significant trends are shown with solid lines ($p<$ 0.05).

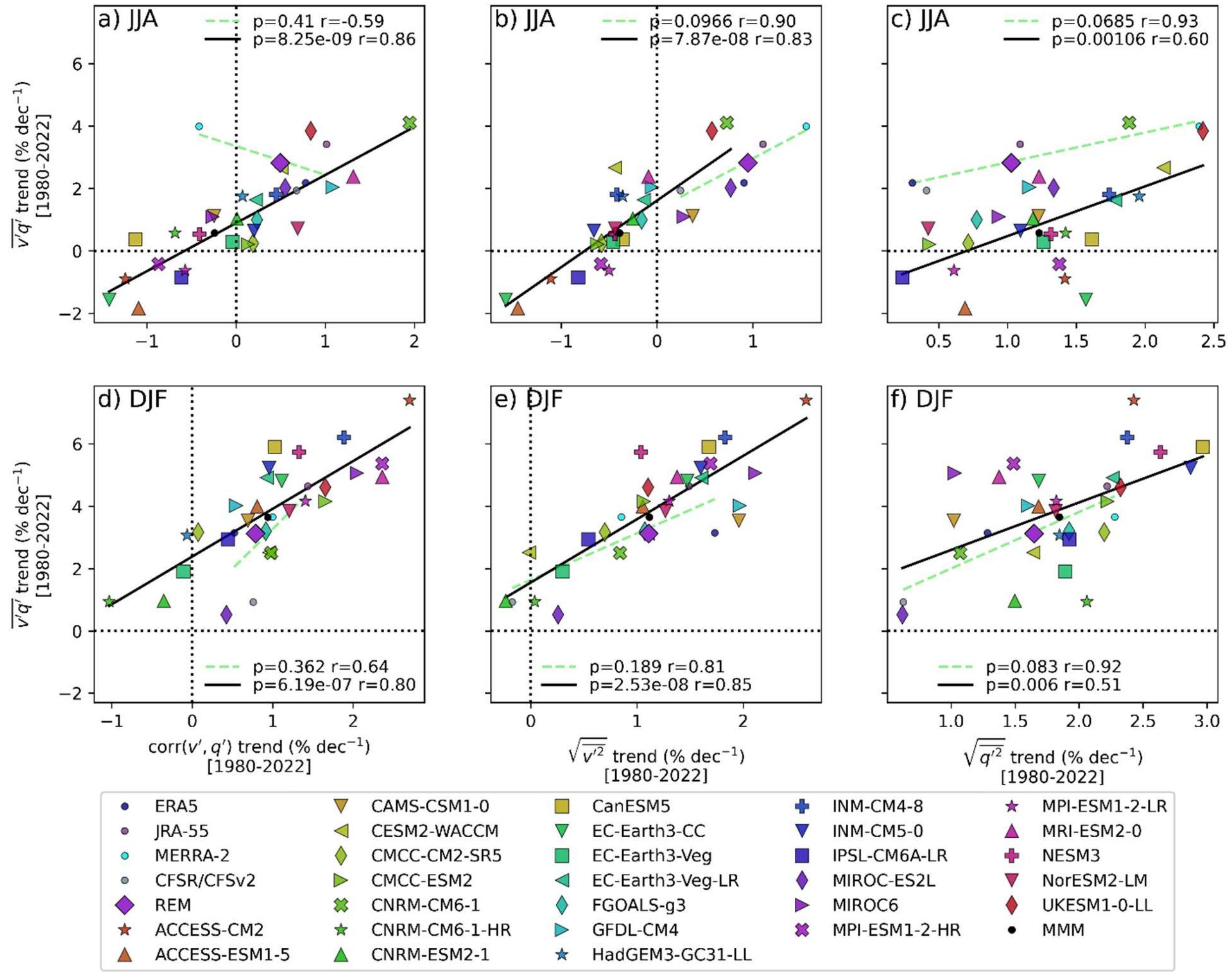

Supplementary Figure 6: Similar to Fig. 7 but for short-term (1980-2022) trends in (a, b, c) JJA and (d, e, f) DJF. Here trends in reanalyses are shown along CMIP6 models. The linear fit is shown separately for reanalyses with green lines.

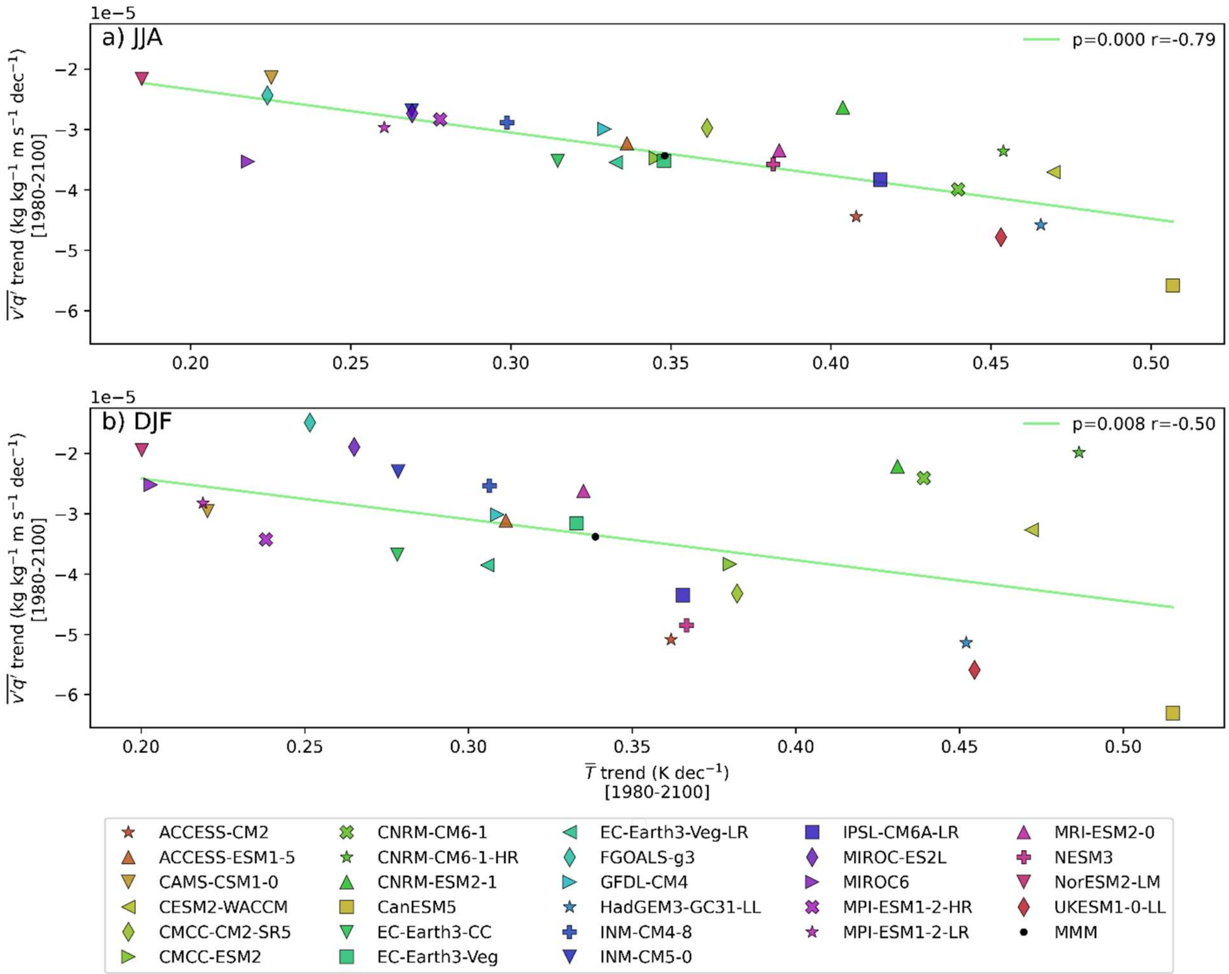

Supplementary Figure 7: Relationship between trends in meridional moisture flux (y-axes) and trends in mean temperature (x-axes) amongst the CMIP6 models at 700 hPa over the Antarctic circle 60°-70°S for the SSP5-8.5 scenario (1980-2100) in (a) JJA and (b) DJF. The *p*-value and correlation on the trend are indicated in each panel. Significant trends are shown with solid lines ($p < 0.05$).
